# Supplementary material for: Pervanadate-induced oxidation relieves autoinhibition of SRC protein tyrosine kinase
Source: Sci Signal. Author manuscript; Available in PMC 2026 Jun 16. (PMC7619190; doi:10.1126/scisignal.ady9437)
Supplement: Supplementary Materials [file EMS214033-supplement-Supplementary_Materials.pdf]

Science Signaling
AAAS

Supplementary Materials for

Pervanadate-induced oxidation relieves autoinhibition of the protein tyrosine kinase SRC

Katie E. Mulholland *et al.*

Corresponding author: Dominic P. Byrne, d.byrne@liverpool.ac.uk;
Hayley J. Sharpe, hayley.sharpe@babraham.ac.uk

Sci. Signal. **19**, eady9437 (2026)
DOI: 10.1126/scisignal.ady9437

The PDF file includes:

Figs. S1 to S6
Legends for movies S1 to S6
Legends for data files S1 to S3
References (*83–86*)

Other Supplementary Material for this manuscript includes the following:

Movies S1 to S6
Data files S1 to S3
MDAR Reproducibility Checklist

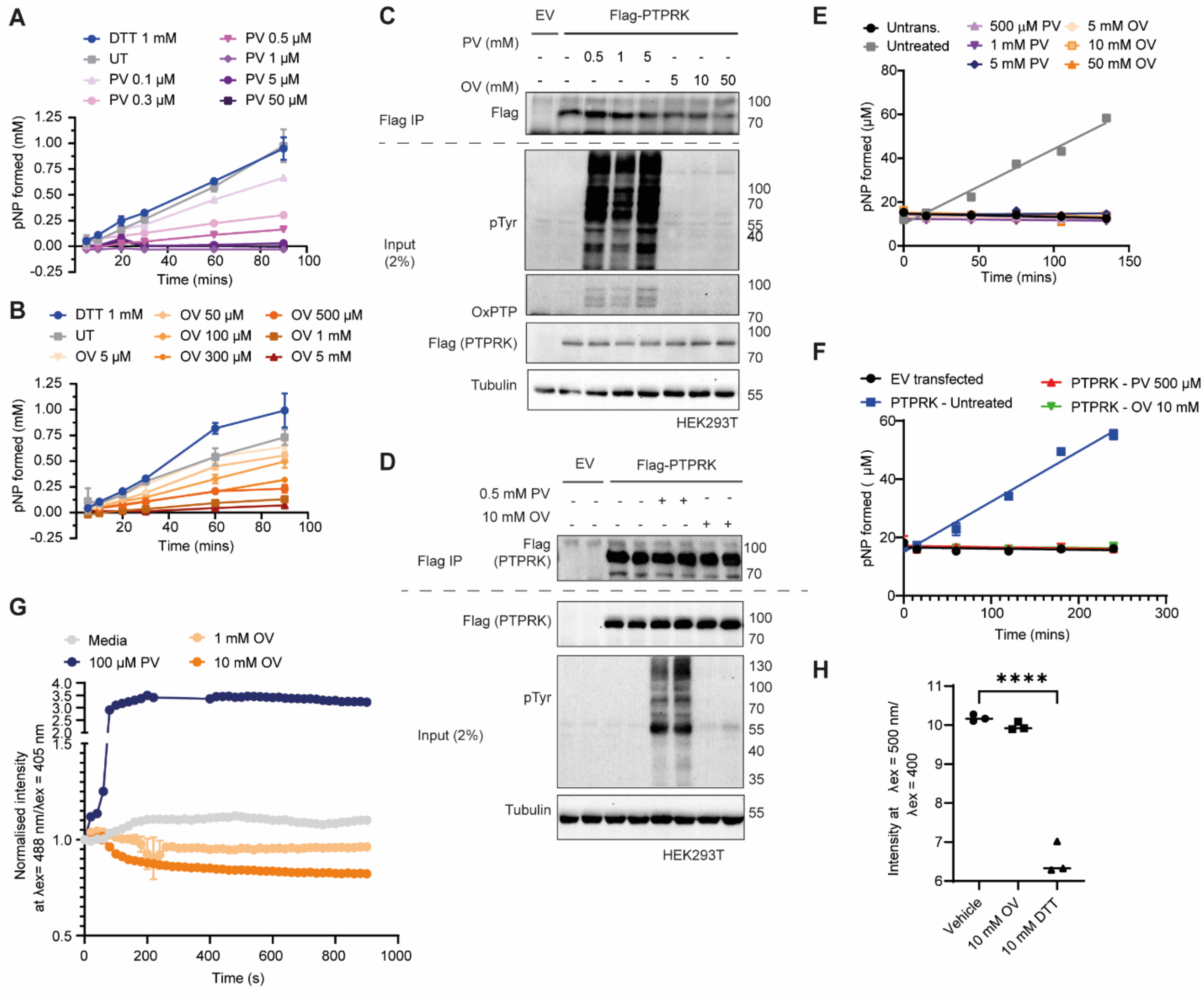

Fig. S1. PV and OV inhibit PTPs, but only PV induces HyPer7 oxidation in cells.
(**A and B**) pNPP phosphatase assays with 0.5 µM recombinant PTPRK intracellular domains with the indicated concentrations of PV (A), OV (B), or DTT. UT = untreated. Mean and SD of 3 independent assays shown. (**C and D**) HEK293T cells were transiently transfected with empty vector (EV) or plasmid for expression of flag-tagged PTPRK intracellular domain (Flag-PTPRK) and treated as shown. Cells were lysed and immunoprecipitated for Flag. Inputs and immunoprecipitates were subject to immunoblot analysis using the indicated antibodies. (**E**) Immunoprecipitates analyzed in C were subjected to a pNPP colorimetric phosphatase assay. (**F**) Immunoprecipitates analyzed in D were subjected to a pNPP colorimetric phosphatase assay. EV= empty vector. (**G**) Quantification of the ratio of the two excitation maxima (F488/405), normalized against initial value, of PM-HyPer7 expressing U2OS cells over a time course, imaged every 20 s. Treatments were added at 60 seconds (arrow). The bars represent mean of one field of view from N=3 independent experiments ± SD. ** $p \le 0.001$. Unpaired t-test at t= 400 s. (**H**) Ratio of the two excitation maxima (F500/400) of bacterially expressed recombinant HyPer7 (2.5 µM) following its treatment with the indicated reagents in vitro. The bars represent the mean of N=3 independent experiments± SD. **** $p \le 0.0001$. The p-value is based on Dunnett's multiple comparisons test of a one-way ANOVA.

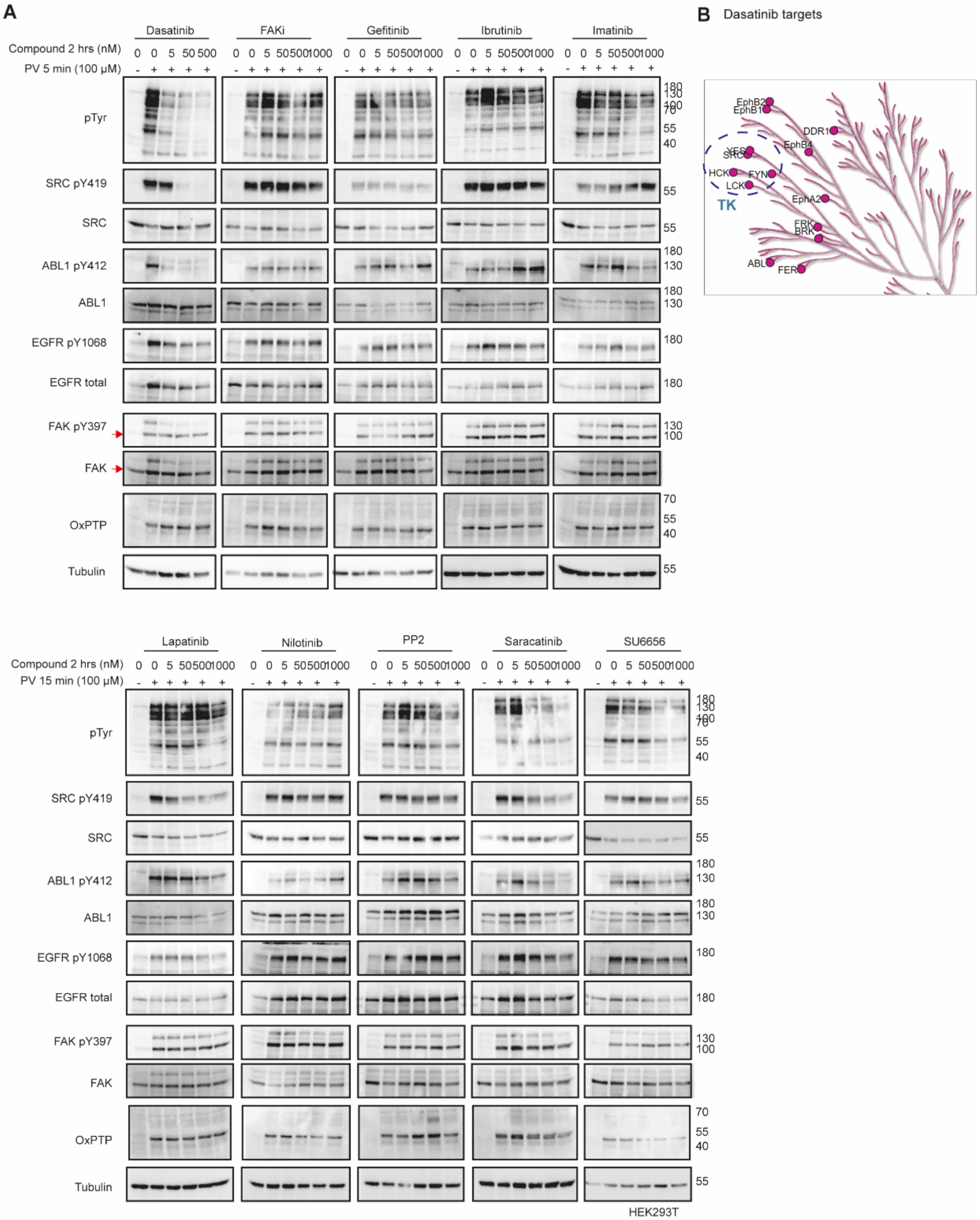

Fig. S2. Kinase inhibitor panel to assess which kinases support the PV response.

(**A**) HEK293T cells were pretreated with the indicated tyrosine kinase inhibitors and concentrations for 2 hours with or without PV (15 minutes) before lysis and immunoblotting with antibodies shown. N = 2. (B) Dasatinib targets based on (*83*) and mapped onto the tyrosine kinase arm of the Human kinome using kinmap (http://kinhub.org/kinmap/). Dashed circle indicates the SRC family kinases.

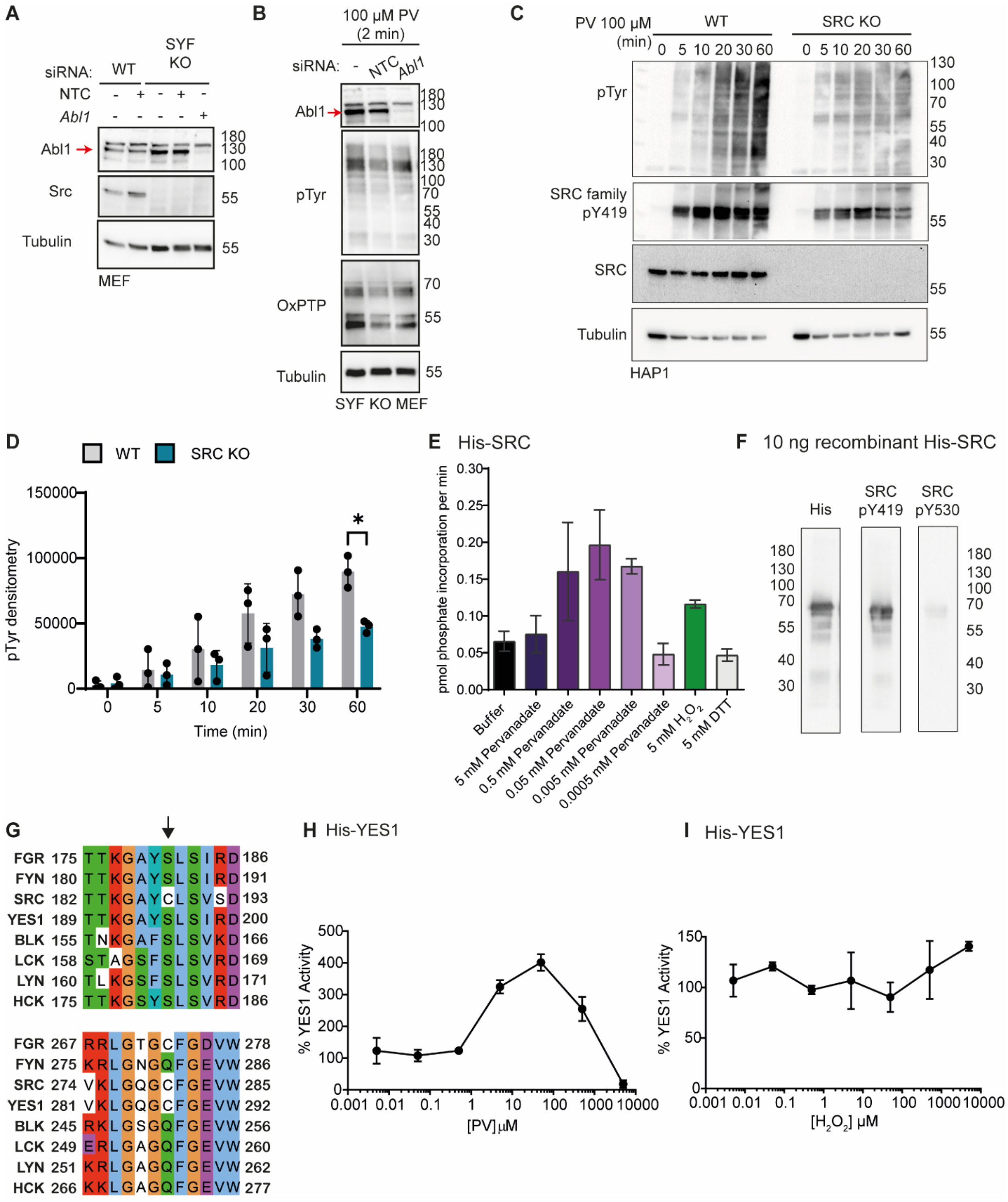
A
siRNA:
WT
SYF KO
NTC
Abl1
Abl1
180
130
100
Src
55
Tubulin
55
MEF
B
100 µM PV (2 min)
siRNA:
NTC
Abl1
Abl1
pTyr
OxPTP
Tubulin
SYF KO MEF
C
PV 100 µM (min)
WT
SRC KO
0 5 10 20 30 60
pTyr
SRC family pY419
SRC
Tubulin
HAP1
D
WT
SRC KO
pTyr densitometry
150000
100000
50000
0
Time (min)
*
E
His-SRC
pmol phosphate incorporation per min
Buffer
5 mM Pervanadate
0.5 mM Pervanadate
0.05 mM Pervanadate
0.005 mM Pervanadate
0.0005 mM Pervanadate
5 mM H2O2
5 mM DTT
F
10 ng recombinant His-SRC
His
SRC pY419
SRC pY530
G
FGR 175 TTKGAYSLSIRD 186
FYN 180 TTKGAYSLSIRD 191
SRC 182 TTKGAYCLSVSD 193
YES1 189 TTKGAYSLSIRD 200
BLK 155 TNKGAFSLSVKD 166
LCK 158 STAGSFSLSVRD 169
LYN 160 TLKGSFSLSVRD 171
HCK 175 TTKGSYSLSVRD 186
FGR 267 RRLGTGCFGDVW 278
FYN 275 KRLGNGQFGEVW 286
SRC 274 VKLGQGCFGEVW 285
YES1 281 VKLGQGCFGEVW 292
BLK 245 RKLGSGQFGEVW 256
LCK 249 ERLGAGQFGEVW 260
LYN 251 KRLGAGQFGEVW 262
HCK 266 KKLGAGQFGEVW 277
H
His-YES1
% YES1 Activity
[PV] µM
I
His-YES1
% YES1 Activity
[H2O2] µM

Fig. S3. Effects of PV and H_2O_2 concentration-dependently activate SRC.
(**A**) WT and SYF KO MEFs were transfected with or without NTC or ABL1 siRNAs, prior to lysis and immunoblotting with indicated antibodies. Red arrow indicates the band for ABL1. N = 1. (**B**) SYF KO MEFs were transfected with or without NTC or ABL1 siRNAs and treated with 100 μM PV for 2 min, prior to lysis and immunoblotting with indicated antibodies. Red arrow indicates the band for ABL1. N = 1. (**C**) Hap1 WT and SRC KO MEFs were treated with 100 μM PV for indicated times prior to lysis and immunoblotting with antibodies shown. N = 3. (**D**) Densitometric quantification of tyrosine phosphorylation from (C). Mean of N=3 independent experiments± SD plotted. P-values based on multiple unpaired t tests after Holm-Sidak correction for multiple comparisons. *p<0.05. (**E**) Change in the rate of SRC-mediated substrate phosphorylation over time (adapted from Fig. 2A). The relative rate of phosphate incorporation (pmol per min) was calculated at each time point and is plotted as a function of time. Means of N=3 independent experiments ± SD are shown. (**F**) Immunoblot analysis of recombinant SRC. N=1. (**G**) Alignment of SFKs highlighting the amino acids surrounding the equivalent of SRC Cys^{188} (upper) and Cys^{280} (lower). Alignment generated using ClustalOmega (*84*) and edited in Jalview (85) with Clustal color scheme. (**H and I**) YES1 (40 ng)-mediated phosphorylation of a specific fluorescent substrate (2 μM) measured in real time in the presence of the indicated concentrations of PV (H) or H_2O_2 (I) following pre-incubation for 10 min. Reactions were initiated with the addition of peptide substrate and ATP, and the rate of peptide phosphorylation was measured after 15- and 25-min assay time. Data is % YES1 activity relative to a buffer only control. Means of N=3 independent experiments ± SD are shown.

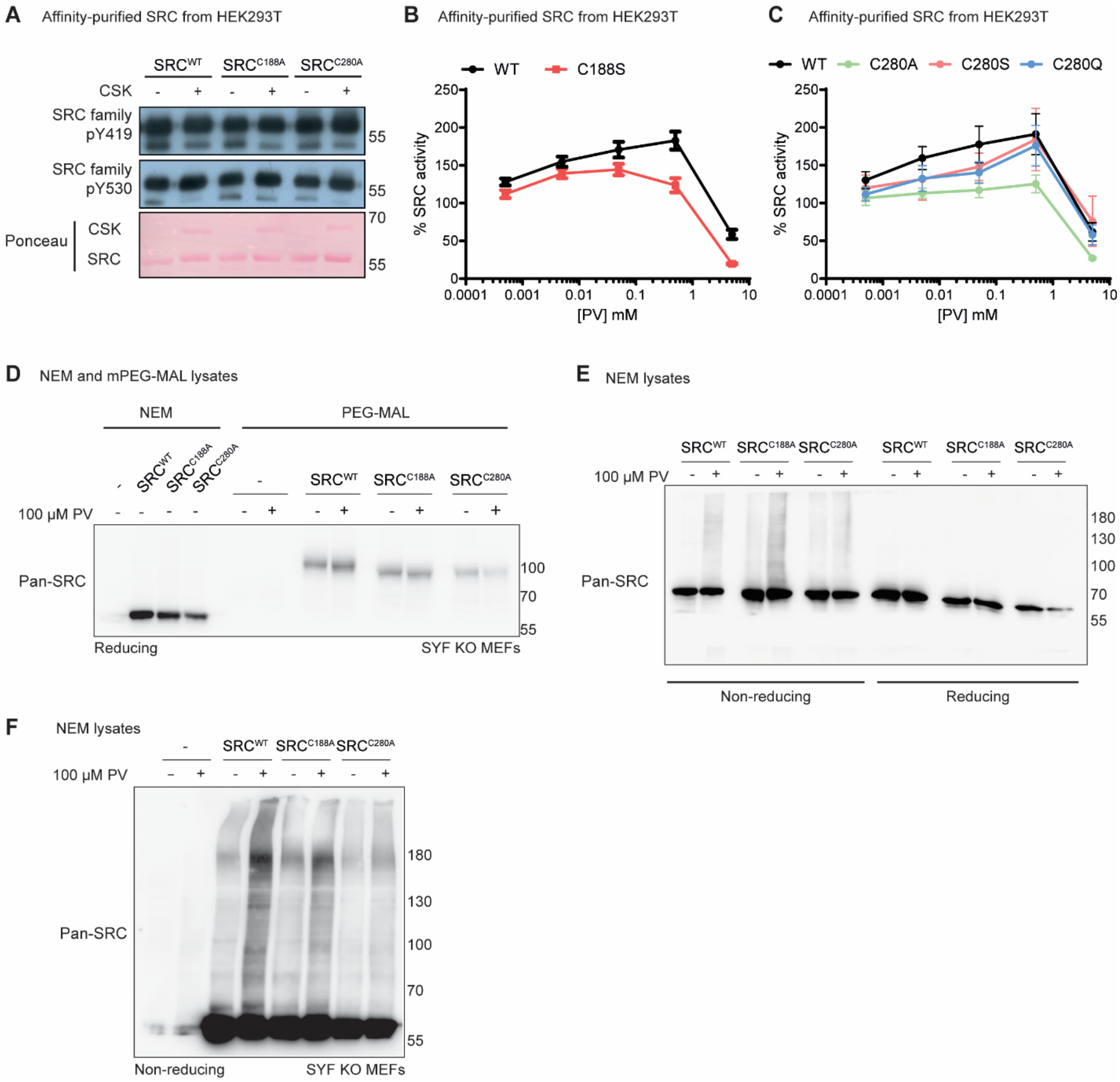
A Affinity-purified SRC from HEK293T
SRCWT SRCC188A SRCC280A
CSK
SRC family pY419
SRC family pY530
Ponceau CSK SRC
55
70
B Affinity-purified SRC from HEK293T
WT C188S
% SRC activity
[PV] mM
C Affinity-purified SRC from HEK293T
WT C280A C280S C280Q
% SRC activity
[PV] mM
D NEM and mPEG-MAL lysates
NEM PEG-MAL
100 µM PV
Pan-SRC
Reducing
SYF KO MEFs
E NEM lysates
100 µM PV
Pan-SRC
Non-reducing
Reducing
F NEM lysates
100 µM PV
Pan-SRC
Non-reducing
SYF KO MEFs

Fig. S4. Characterization of SRC cysteine mutants.

(**A**) Immunoblot analysis of indicated purified recombinant proteins. Loading is shown with ponceau. N = 1. (**B**) Concentration-response curves for activities of affinity-purified WT, C188S SRC incubated with the indicated concentrations of PV for 10 mins prior to the assay. Phosphorylation of the fluorescent peptide substrate was measured after 15 min. Data shown are % SRC activity compared to a buffer control. Means of N=3 ±SD are shown. **(C)** Concentration-response curves for activities of affinity-purified WT, C280A, and C280S and C280Q SRC incubated with the indicated concentrations of PV for 10 mins prior to the assay. Phosphorylation of the fluorescent peptide substrate was measured after 15 min. Data shown are % SRC activity compared to a buffer control. Means of N=3 ±SD are shown. (**D**) Immunoblot analysis of cell lysates treated with mPEG2k-MAL following reducing SDS-PAGE. Image representative of N=3 independent experiments. (**E**) Immunoblot analysis of cell lysates treated with NEM following non-reducing and reducing SDS-PAGE. Image representative of N=2 independent experiments. (**F**) Immunoblot analysis of cell lysates treated with NEM following non-reducing SDS-PAGE. Image representative of N=2 independent experiments.

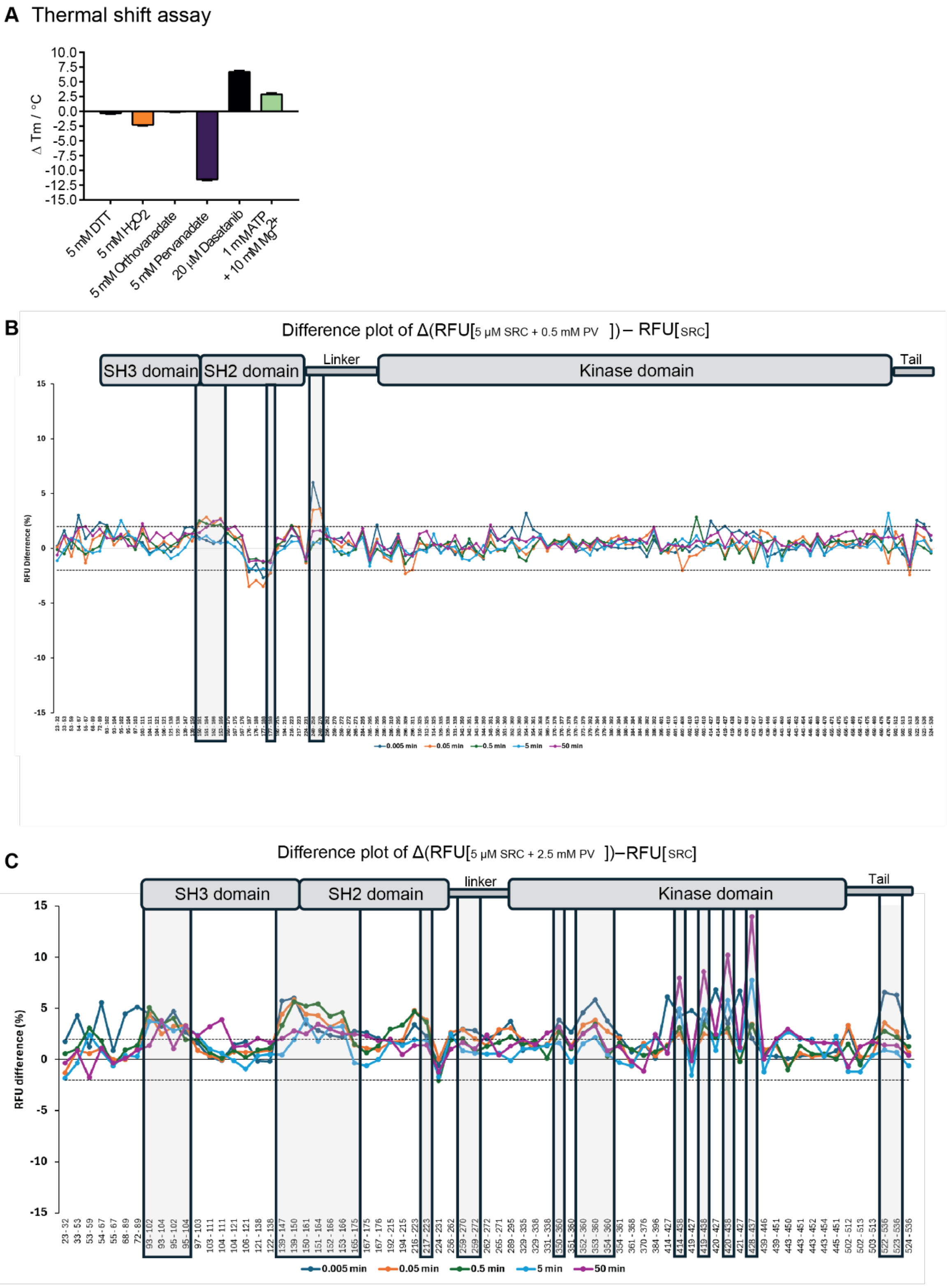
A Thermal shift assay
Δ Tm / °C
10.0
7.5
5.0
2.5
0.0
-2.5
-5.0
-7.5
-10.0
-12.5
-15.0
5 mM DTT
5 mM H2O2
5 mM Orthovanadate
5 mM Pervanadate
20 μM Dasatanib
1 mMATP + 10 mM Mg2+
B
Difference plot of Δ(RFU[5 μM SRC + 0.5 mM PV]) – RFU[SRC]
SH3 domain
SH2 domain
Linker
Kinase domain
Tail
RFU Difference (%)
15
10
5
0
-5
-10
-15
0.005 min
0.05 min
0.5 min
5 min
50 min
C
Difference plot of Δ(RFU[5 μM SRC + 2.5 mM PV])–RFU[SRC]
SH3 domain
SH2 domain
linker
Kinase domain
Tail
RFU difference (%)
15
10
5
0
-5
-10
-15
0.005 min
0.05 min
0.5 min
5 min
50 min

Fig. S5. Differential relative fractional uptake across SRC peptides following HDX-MS. (**A**) Thermal shift analysis of 2.5 μM purified full-length SRC treated as indicated. Shifts relative to untreated protein from N=2 independent assays are shown. (**B**) Relative fractional uptake (RFU) difference plots are represented for HDX-MS analysis of SRC for 0.5 mM PV versus untreated control. RFU differences are depicted for 0.005, 0.05, 0.5, 5 and 50 minutes of deuteration. Framed peptides represent the identified peptides for which the difference of deuterium incorporation has been statistically validated for at least two timepoints. (**C**) Relative fractional uptake difference plots are represented for HDX-MS analysis of SRC for 0.5 mM PV versus untreated control. RFU differences are depicted for 0.005, 0.05, 0.5, 5 and 50 minutes of deuteration. Framed peptides represent the identified peptides for which the difference of deuterium incorporation has been statistically validated for at least two timepoints. For (B) and (C), N = 3.

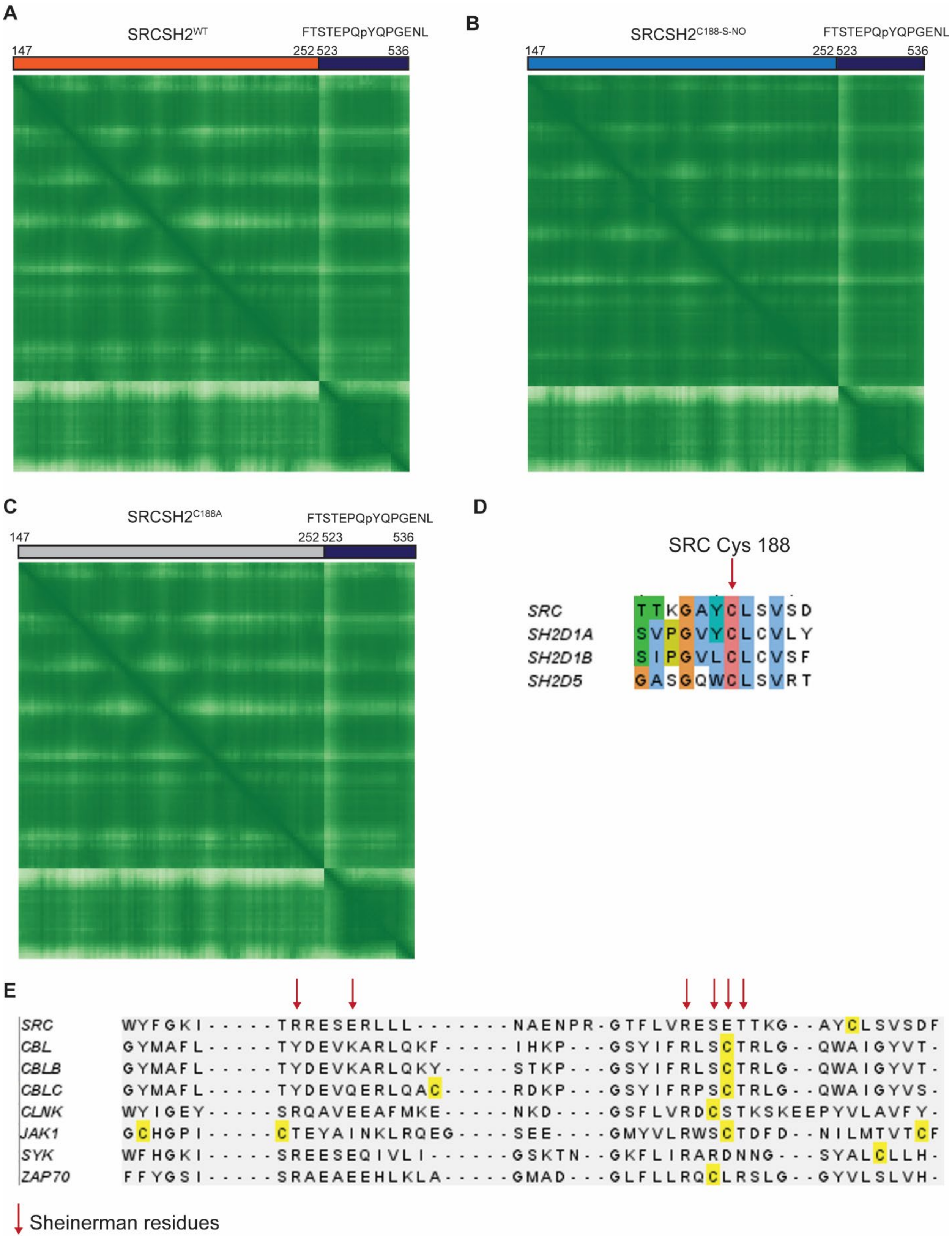

A
SRCSH2WT
FTSTEPQpYQPGENL
147
252 523
536
B
SRCSH2C188-S-NO
FTSTEPQpYQPGENL
147
252 523
536
C
SRCSH2C188A
FTSTEPQpYQPGENL
147
252 523
536
D
SRC Cys 188
SRC TTKGAYCLSVSD
SH2D1A SVPGVYCLCVLY
SH2D1B SIPGVLCLCVSF
SH2D5 GASGQWCLSVRT
E
SRC WYFGKI-----TRRESERLLL-------NAENPR-GTFLVRESETTKG--AYCLSVSDF
CBL GYMAFL-----TYDEVKARLQKF-----IHKP---GSYIFRLSCTRLG--QWAIGYVT-
CBLB GYMAFL-----TYDEVKARLQKY-----STKP---GSYIFRLSCTRLG--QWAIGYVT-
CBLC GYMAFL-----TYDEVQERLQAC-----RDKP---GSYIFRPSCTRLG--QWAIGYVS-
CLNK WYIGEY-----SRQAVEEAFMKE-----NKD----GSFLVRDCSTKSKEEPYVLAVFY-
JAK1 GCHGPI-----CTEYAINKLRQEG----SEE----GMYVLRWSCTDFD--NILMTVTCF
SYK WFHGKI-----SREESEQIVLI------GSKTN--GKFLIRARDNNG---SYALCLLH-
ZAP70 FFYGSI-----SRAEAEEHLKLA-----GMAD---GLFLLRQCLRSLG--GYVLSLVH-
Sheinerman residues

Fig. S6. AlphaFold3 error plots corresponding to models shown in Fig. 4C.
(**A to C**) AF3 error plot for WT SRC-SH2 (A), SRC-SH2^{C188A} (B), and SRC-SH2^{C188A} (C) interaction with the SRC C-terminal phosphopeptide. Darker green indicates lower Expected Position Error (Ångstroms). (**D**) Clustal alignment of region spanning SRC Cys^{188} with equivalent regions in the SH2 domains of SH2D1A, SH2D1B, SH2D5. Edited in Jalview (85). (**E**) Alignment highlighting cysteine residues within the pTyr-binding pocket of SH2 domains from indicated proteins. Alignments derived from SH2DB (86). Edited in Jalview. Sheinerman residues highlighted by red arrow (86).

Movie S1. Effect of media mock treatment on redox homeostasis at the plasma membrane of U2OS cells. U2OS cells stably expressing PM-HyPer7 were imaged using total internal reflection fluorescence (TIRF) microscopy. After 60s cells were treated with media as a mock treatment. Representative of N=3 experimental replicates performed on different days and quantified in Fig. 1D.

Movie S2. Effect of PV treatment on redox homeostasis at the plasma membrane of U2OS cells. U2OS cells stably expressing PM-HyPer7 were imaged using TIRF microscopy. After 60s cells were treated with 100 μM PV. Representative of N=3 experimental replicates performed on different days and quantified in Fig. 1D.

Movie S3. Timelapse of SYF KO MEF cells in culture. Cells were plated and imaged for 6 days then analyzed using an IncuCyte system. Related to Fig. 5B.

Movie S4. Timelapse of SYF KO + SRC MEF cells in culture. Cells were plated and imaged for 6 days then analyzed using an IncuCyte system. Related to Fig. 5B.

Movie S5. Timelapse of SYF KO + SRC^{C188A} MEF cells in culture. Cells were plated and imaged for 6 days then analyzed using an IncuCyte system. Related to Fig. 5B.

Movie S6. Timelapse of SYF KO + SRC^{C280A} MEF cells in culture. Cells were plated and imaged for 6 days then analyzed using an IncuCyte system. Related to Fig. 5B.

Data file S1. Raw Mass spectrometry data for cysteine oxidation of affinity purified SRC shown in Fig. 2E and Fig. 2F. These data are provided in an Excel file.

Data file S2. HDX-MS Information. Details about the HDX-MS experiments are provided in an Excel file.

Data file S3. Primer sequences used for molecular cloning and siRNA details. Primer sequences are provided in an Excel file.
